# Retrospective observational study of emergency admission, readmission and the ‘weekend effect’

**DOI:** 10.1136/bmjopen-2016-012493

**Published:** 2017-03-02

**Authors:** Ivy Shiue, Peter McMeekin, Christopher Price

**Affiliations:** 1 Faculty of Health and Life Sciences, Northumbria University, Newcastle upon Tyne, UK; 2 Faculty of Health and Life Sciences, Northumbria University, Newcastle upon Tyne, UK; 3 Institute of Neuroscience, Newcastle University, Newcastle upon Tyne, UK

**Keywords:** ACCIDENT & EMERGENCY MEDICINE, EPIDEMIOLOGY, HEALTH SERVICES ADMINISTRATION & MANAGEMENT

## Abstract

**Objectives:**

Excess mortality following weekend hospital admission has been observed but not explained. As readmissions have greater age, comorbidity and social deprivation, outcomes following emergency index admission and readmission were examined for temporal and demographic associations to confirm whether weekend readmissions contribute towards excess mortality.

**Design:**

A retrospective observational study. Individual patient Hospital Episode Statistics were linked and 2 categories created: index admissions (not within 60 days of discharge from an emergency hospitalisation) and readmissions (within 60 days of discharge from an emergency hospitalisation). Logistic regression examined associations between admission category, weekend and weekday mortality, age, gender, season, comorbidity and social deprivation.

**Setting:**

A single acute National Health Service (NHS) trust serving a population of 550 000 via 3 emergency departments.

**Participants:**

Emergency admissions between 1 January 2010 and 31 March 2015.

**Outcome measure:**

All-cause 30-day mortality.

**Results:**

Over 5 years there were 128 966 index admissions (74.7% weekday/25.3% weekend) and 20 030 readmissions (74.9% weekday/25.1% weekend). Adjusted 30-day death rates for weekday/weekend admissions were 6.93%/7.04% for index cases and 12.26%/13.27% for readmissions. Weekend readmissions had a higher mortality risk relative to weekday readmissions (OR 1.10 (95% CI 1.01 to 1.20)) without differences in comorbidity or deprivation. Weekend index admissions did not have a significantly increased mortality risk (OR 1.04 (95% CI 0.98 to 1.11)) but deaths which did occur were associated with lower deprivation (OR 1.24 (95% CI 1.11 to 1.38)) and an absence of comorbidities (OR 1.17 (1.02 to 1.34)).

**Conclusions:**

Associations with emergency hospitalisation were not identical for index admissions and readmissions. Further research is needed to confirm what factors are responsible for the ‘weekend effect’.

**Strengths and limitations of the study**

- Previous studies have been unable to examine the impact of readmissions due to the challenge of data linkage for individual patients.
- The data set was a continuous aggregate over 5 years within a stable service, thereby reducing the impact of structural changes on emergency admissions.
- The primary outcome (death) was captured at 30 days, whether or not the patient was in hospital or the community.

- We did not consider the impact of multiple readmissions during 60 days as the small size of the corresponding subgroups would have prevented meaningful analysis.
- The specific circumstances of this single-centre cohort should be considered when determining the wider relevance.
- It is possible that a larger cohort might have also shown a statistically significant weekend effect for index admissions, but it is notable that the association was already evident in the smaller readmission group.

## Introduction

Descriptions of survival in large unselected hospital populations have generally reported excess deaths specifically related to weekend admission,^1–6^ although this has not been universal.^7^ ^8^ The mechanism of this ‘weekend effect’ remains controversial, but could have important implications for the organisation and resourcing of emergency medical services. It has been proposed that the size and expertise of the weekend hospital workforce influences health outcomes,^1^ ^3^ ^6^ ^9^ but explanations are obscured by the use of large administrative data sets from different geographical and service settings, which are not linked to information about illness severity or the availability of clinicians and treatments inside and outside of secondary care.

Readmissions within 30 days have been observed to account for 7% of National Health Service (NHS) discharges and are more likely following recent emergency hospitalisation.^10–12^ Compared with index admissions, readmissions have characteristics associated with a higher risk of death, including greater age, comorbidity and social deprivation.^11^ ^12^ As community services may have less flexibility in their response to unexpected decompensation of health and social status over the weekend, we hypothesised in advance of data analysis that readmissions could contribute disproportionately towards weekend mortality through an increased risk of: (1) hospitalisation at a weekend; (2) death relative to index admissions and (3) death relative to weekday readmissions. If correct, this would infer that previous attempts at case-mix correction were inadequate,^1–6^ and the weekend effect might also reflect an interaction between community and outpatient services, failure of initial treatments and advancing disease progression following an index admission.

Owing to the challenge of case linkage, previous reports have considered individual admissions rather than patient-level analysis, and were unable to examine the relevance of a second hospitalisation soon after discharge. Using linked patient-level data to isolate index admissions from readmissions, we examined associations with the outcome from emergency hospitalisation at a single large NHS healthcare provider over 5 years in order to confirm or refute the presence of a weekend effect and describe relevant sociodemographic characteristics.

## Methods

### Study setting and population

Unscheduled admissions between 1 January 2010 and 31 March 2015 were identified in Hospital Episode Statistics from a single acute hospital trust serving a population of 550 000 via three emergency departments (EDs; table 1).^13^ The cohort included only ED admissions by ambulance, general practitioner (GP) or self-presentation but did not include hospital transfers or GP admissions directly to an inpatient area. The standard route for unscheduled primary care referrals was via ED. During this interval, unselected patients were admitted to each ED without prehospital redirection, apart from those with suspected myocardial infarction who were diverted to a regional cardiology centre according to ambulance service protocol. Twice daily consultant ward rounds took place on each admissions unit throughout the week and a critical care outreach team was always available.

**Table 1:** Service coverage across three ED sites

ED	Total population served	People resident per sq mile/sq km (average)	Description
A	235 000	6242/2401	Uniform urban and suburban city population all within 10 miles of the ED
B	255 000	233/603	Majority of population in 5 towns between 1 and 50 miles from the ED
C	60 000	70/27	Majority of population within 5 miles of the ED in a single town, rest widely dispersed in a rural setting

ED, emergency department.

### Variables and analyses

Two admission categories were created from linked individual patient records: index admissions (not within 60 days of discharge from an emergency hospitalisation) and readmissions (within 60 days of discharge from an emergency hospitalisation). An interval of 60 days was chosen to allow sufficient time for any medical review in outpatients or the community that might be triggered by an index event, and it was assumed that another admission after 60 days was unrelated. Over the 5 years, patients could appear more than once in the index or readmission data set according to the timing of their attendances. If patients were readmitted more than once within 60 days of discharge from an index event, only the first readmission was included in the analysis.

Weekend admissions were defined as arrival at ED any time from 00:00 on Saturday morning to 24:00 on Sunday night. There was no correction for bank holidays. The Charlson Comorbidity Index (CCI)^14^ was calculated for each admission and the Index of Multiple Deprivation Score (IMDS)^15^ was derived from lower super output areas. The main outcome was all-cause 30-day mortality for patients with at least 1-day length of stay, although patients who died on the day of admission were still included in the numerator and denominator. The hospital administration system is routinely updated with the dates of inpatient and community deaths. Crude death rates were adjusted for age and gender. Descriptive statistics and χ^2^ test were used to describe and compare binary variables. Logistic regression was used to determine any association between admission at a weekend, demographic covariates and death within 30 days (the response variable).^4^ ^9^ In addition to deprivation and comorbidities, a five knot spline approach examined the effect of age (8, 52, 70, 81 and 91 years) and season (16 January, 31 March, 18 June, 17 September and 15 December).^16^

To allow for heteroscedasticity, SEs were estimated after clustering International Classification of Diseases (ICD)10 diagnoses into groups defined by Clinical Classifications Software (CCS).^17^ To explore case-mix variations, a second regression analysis examined associations between death following weekend hospitalisation, admission category (index vs readmission), IMDS (top 25% vs bottom 75%) and CCI (0 vs at least 1). Analyses were carried out using SPSS V.22.0 (IBM; more details via http://www-01.ibm.com/software/analytics/spss/) and STATA V.14.0 (STATA, College Station, Texas, USA; more details via http://www.stata.com/) software.

### Patient involvement

Members of the public were not involved in the study concept or design.

## Results

Over 5 years there were 148 996 emergency admissions, comprising 128 966 index cases (74.7% weekday and 25.3% weekend arrival) and 20 030 readmissions within 60 days (74.9% weekday and 25.1% weekend arrival), that is, a readmission rate of 13.4% with the same weekday/weekend distribution as index admissions (p=0.54). By day 30 there were 11 476 (7.7%) deaths. Table 2 shows the distribution of demographic characteristics and deaths according to admission category and timing. Readmissions were older, with higher levels of social deprivation and comorbidity. Readmissions had a significantly greater risk of dying than index admissions. Although there was a relative increase in weekend death rate for readmissions compared with index admissions (7.4% vs 1.6%), there was no significant increase in the 30-day death rates following weekend admission.

**Table 2:** Characteristics of the admission groups

	All admissions		Index admissions only		Readmissions only	
	Monday to Friday	Saturday+Sunday	Monday to Friday	Saturday+Sunday	Monday to Friday	Saturday+Sunday
Number	111 388	37 608	96 379	32 587	15 009	5021
Age (median (IQR))	70 (49, 82)	70 (47, 82)	69 (47, 81)	65 (45, 81)	75 (59, 84)	76 (62, 84)
Male (%)	44.9	44.7	45.0	44.6	44.3	45.6
IMDS (median (IQR))	21.6 (12.1, 34.6)	21.7 (12.1, 34.6)	21.5 (12.0, 34.6)	21.6 (12.1, 34.6)	21.8 (12.4, 35.2)	22.0 (12.8, 35.2)
CCI (median (IQR))	1 (0, 2)	1 (0, 2)	1 (0, 2)	1 (0, 2)	1 (0, 3)	1 (0, 3)
Number of deaths by day 30	8521	2975	6679	2291	1840	666
Crude 30-day death rate (%)	7.65	7.91	6.93	7.03	12.26	13.27
Adjusted 30-day death rate (% (95% CI))	7.66 (7.5 to 7.8)	7.90 (7.6 to 8.2)	6.93 (6.8 to 7.1)	7.04 (6.8 to 7.3)	12.36 (11.8 to 12.9)	13.27 (12.4 to 14.2)

CCI, Charlson Comorbidity Index; IMDS, Index of Multiple Deprivation Score.

The logistic regression output (table 3) indicated an overall increased risk of dying associated with male gender, very young or old age, increasing comorbidities and greater deprivation. There was a mild seasonal effect. Online supplementary table S1 shows that using a 30-day readmission definition resulted in a very similar output, but a few associations lost statistical significance because of the smaller number of cases.

**Table 3:** Associations with 30-day mortality by admission group in 2010–2015

	All admissions		Index admissions only		Readmissions only	
	OR (95% CI)	p Value	OR (95% CI)	p Value	OR (95% CI)	p Value
Male	1.32 (1.19 to 1.47)	<0.001	1.29 (1.14 to 1.46)	<0.001	1.42 (1.25 to 1.62)	<0.001
CCI	1.13 (1.08 to 1.18)	<0.001	1.12 (1.08 to 1.18)	<0.001	1.10 (1.05 to 1.15)	<0.001
IMDS	1.01 (1.00 to 1.01)	<0.001	1.01 (1.00 to 1.01)	<0.001	1.00 (1.00 to 1.01)	0.035
Weekend admission	1.06 (1.01 to 1.11)	0.023	1.04 (0.98 to 1.11)	0.165	1.10 (1.01 to 1.20)	0.028
Age 1 (youngest)	1.10 (1.08 to 1.13)	<0.001	1.10 (1.07 to 1.13)	<0.001	1.10 (1.05 to 1.16)	<0.001
Age 2	0.97 (0.94 to 0.99)	0.038	0.97 (0.94 to 1.00)	0.066	0.96 (0.90 to 1.02)	0.189
Age 3	0.91 (0.72 to 1.15)	0.420	0.93 (0.73 to 1.18)	0.534	0.88 (0.58 to 1.33)	0.540
Age 4 (oldest)	2.62 (1.47 to 4.69)	0.001	2.46 (1.36 to 4.44)	0.003	3.13 (1.03 to 9.54)	0.044
Date 1 (early year)	0.99 (0.99 to 0.99)	<0.001	0.99 (0.99 to 0.99)	0.002	0.99 (0.99 to 0.99)	0.015
Date 2	1.01 (1.00 to 1.03)	0.006	1.01 (1.00 to 1.02)	0.022	1.02 (0.99 to 1.04)	0.070
Date 3	0.96 (0.94 to 0.99)	0.008	0.97 (0.94 to 0.99)	0.026	0.96 (0.91 to 1.01)	0.087
Date 4 (late year)	1.05 (1.02 to 1.08)	0.003	1.05 (1.01 to 1.08)	0.010	1.05 (0.99 to 1.10)	0.096

Age spline knots: 8, 52, 70, 81 and 91 years; date spline knots: 16 January, 31 March, 18 June, 17 September and 15 December.

CCI, Charlson Comorbidity Index; IMDS, Index of Multiple Deprivation Score.

10.1136/bmjopen-2016-012493.supp1 supplementary table Associated factors with 30-day mortality by admission groups according to readmission at day 30

Table 3 shows that weekend admission was associated with a small increased risk of death overall (OR 1.06 (95% CI 1.01 to 1.11)). This was statistically significant for readmissions (OR 1.10 (95% CI 1.01 to 1.20)) but not for index admissions (OR 1.04 (95% CI 0.98 to 1.11)), suggesting that admission category should be a data field reported by other ‘weekend effect’ studies. Further regression analysis (table 4) indicated that there was an increased risk of death for weekend index admissions with the least social deprivation (n=32 742; OR 1.24 (95% CI 1.11 to 1.38)) and without comorbidities (n=70 299; OR 1.17 (95% CI 1.02 to 1.34)). There was no additional interaction between these characteristics. Deaths following weekend readmission did not differ in IMDS or CCI when compared with readmissions between Monday and Friday.

**Table 4:** Interactions between 30-day death, social deprivation and comorbidities for weekend admissions

	Risk of 30-day death after a weekend admission			
	Index admissions only		Readmissions only	
	OR (95% CI)	p Value	OR (95% CI)	p Value
No comorbidities (CCI=0)	1.17 (1.02 to 1.34)	0.023	1.05 (0.85 to 1.30)	0.638
Least social deprivation (IMDS top quartile)	1.24 (1.11 to 1.38)	<0.001	0.95 (0.72 to 1.25)	0.725
No comorbidities × least social deprivation	0.90 (0.72 to 1.13)	0.380	1.09 (0.65 to 1.85)	0.726

No comorbidities was defined as a CCI of zero; least social deprivation represents the top quartile of the IMDS range.

CCI, Charlson Comorbidity Index; IMDS, Index of Multiple Deprivation Score.

## Discussion

During 5 years of emergency activity across three EDs there was no significant increase in the overall adjusted 30-day death rate following weekend admission, but a small ‘weekend effect’ was identified by multivariable analysis. The readmission rate of 13.4% within 60 days of discharge was consistent with a previously reported rate of 12.9% at 28 days derived from English Hospital Episode Statistics for acute medical admissions.^6^ Readmissions had a higher death rate, but the weekday/weekend distribution was similar to that of index admissions. However, multivariable analysis indicated that weekend readmission was associated with a small increase in the risk of death compared with weekday readmission. This was not explained by comorbidities or social deprivation.

The size of the association found between weekend readmission and mortality was similar to that of previous studies. From analysis of 4 317 866 emergency admissions across England during 2005–2006, Aylin *et al*^1^ reported an overall adjusted odds of death for weekend hospitalisation of 1.10 (95% CI 1.08 to 1.11) compared with patients admitted during a weekday. For 14 217 640 unselected English NHS admissions during 2009–2010, Freemantle *et al*^4^ reported a HR for death following hospitalisation on a Saturday of 1.11 (95% CI 1.09 to 1.13) and for a Sunday of 1.16 (95% CI 1.14 to 1.18). An updated analysis of 14 818 374 admissions during 2013–2014 showed similar ratios of 1.10 (95% CI 1.08 to 1.11) for Saturday and 1.15 (95% CI 1.14 to 1.17) for Sunday.^9^

It has been suggested that the excess mortality observed following weekend admission is due to the size of the hospital workforce.^1^ ^4^ ^6^ ^9^ This seems less likely in our cohort as the effect was not the same within each admission category. One possible explanation is that the higher mortality rate of readmissions implies greater illness severity, which could amplify the impact of any weekend shortfall in initial clinical availability. However, a survival difference between index admission and readmission might also reflect variations in case-mix and overall service provision including care in between hospital episodes. Since readmission was defined as a second hospitalisation within 60 days of an unscheduled index discharge, the outcome could be related to medical and social stability when initially leaving hospital, the effectiveness and complications of treatment started during the initial admission, and the ability of community and outpatient services to compensate for individuals with rapidly progressive and fluctuating states. Risk factors for readmission have previously been described including age, prior emergency discharge, IMDS and major health conditions (eg, from the CCI)^12^ and combined into predictive models with c-statistics ranging from 0.50 to 0.72.^18^ As readmission cohorts display distinct demographic and service-user profiles it would appear simplistic to suggest that the size of the workforce only on the day of readmission is solely responsible for excess mortality. In population-level studies, the CCI has been used to reflect illness burden, but it is insensitive to short-term health changes and an additional association between admission category and survivorship strongly suggests that measures of acute illness severity and clinical care beyond the day of admission are necessary to understand the ‘weekend effect’.

The influence of case-mix on outcome following weekend hospitalisation was also implied by the observation that index deaths were associated with reversal of typical demographic trends,^19^ ^20^ and the risks associated with low social deprivation and an absence of comorbidities were of greater magnitude than the timing of admission. The mechanism is unclear, but as these groups are less likely to include frequent service users, patients might present directly to ED at later stages of an acute illness. Their prognosis may be poorer relative to patients with milder exacerbations of chronic conditions who are in regular contact with primary care and consequently are more likely to be admitted in larger numbers on a weekday following GP review. A mixed-methods approach would be helpful to further investigate this finding, including qualitative interviews with purposive sampling of patients, the addition of physiological data to indicate initial illness severity, cross-referencing with outpatient attendances and linkage to primary care records to observe the timing of any contacts leading up to admission.

As the study focussed on emergency admissions, it is not surprising that the overall mortality rate of 7.7% was higher than cohorts which have also included scheduled care.^4^ ^9^ ^19^ ^20^ It was consistent with a previous in-hospital mortality report of 7.96% among 15 594 emergency medical admissions to a single centre.^8^ Examinations of unscheduled activity across multiple acute hospital trusts in England have reported an overall crude mortality rate of 5.0%^1^ and an adjusted case fatality rate of 4.3% (range 2.5–6.4%).^6^ However, these inpatient population studies included groups in the denominator which could have a lower short-term risk of death such as hospital transfers and direct ward admissions from primary care, and did not count deaths occurring in the community after discharge.^21^ As there are many factors which could influence inpatient mortality, it is important that future reports clearly define the patient groups contributing towards outcomes in order to allow fair comparison and identify mechanisms that may be responsible for temporal trends.

Previous studies have been unable to examine the impact of readmissions due to the challenge of data linkage for individual patients. We did not consider the impact of multiple readmissions during 60 days as the small size of the corresponding subgroups would have prevented meaningful analysis. The specific circumstances of this study cohort should be considered when determining the wider relevance, that is, the results describe outcomes from a single large acute care provider organisation with consistent daily levels of senior medical cover to review emergency admissions, thereby reducing any potential workforce-related weekend effect.^6^ Although the analysis attempted to correct for case-mix effects, due to survivorship bias and the greater illness severity associated with readmission it may have been easier to detect associations with mortality. It is possible that a larger cohort might have also shown a statistically significant weekend effect for index admissions, but it is notable that the association was already evident in the smaller readmission group. The data set was an aggregate over 5 years, but no significant change in service configuration or admission processes occurred during that time. In order to confirm the associations found and further explore the underlying mechanisms, linkage would be required between primary, secondary and social care data, including reliable indicators of workforce availability and service usage. If behavioural factors and admission category do lead to the clustering of new inpatients with greater illness severity at weekends, interventions could seek to improve patient education, reduce premature discharges, enhance communication between secondary and primary care, and develop alternatives to emergency admission when a patient deteriorates in the community, for example, a more rapid palliative or elderly care response.

In summary, a weak association was identified between 30-day mortality and emergency admission at a weekend, which was statistically significant only for readmissions. A reversal of the usual comorbidity and deprivation trends was observed for death following a weekend index admission, which has not previously been reported and could reflect heterogeneity in health-related behaviours and opportunities for admission. These findings suggest that the ‘weekend effect’ reflects case-mix variations and a whole system responsiveness which cannot be demonstrated through Hospital Episode Statistics alone. Readmissions are likely to have additional complexities from clinical and service perspectives, and the outcome may reflect community as well as hospital responsiveness. We recommend that detailed case-mix characteristics should be considered when examining the potential impact of service provision on patient outcomes.
