# Supplementary material for: Retrospective observational study of emergency admission, readmission and the ‘weekend effect’
Source: BMJ Open. 2017 Mar 2;7(3):e012493. doi: 10.1136/bmjopen-2016-012493 (PMC5353295; doi:10.1136/bmjopen-2016-012493)
Supplement: supplementary table — Associated factors with 30-day mortality by admission groups according to readmission at day 30 [file bmjopen-2016-012493supp_table.pdf]

Supplemental Table 1 Associated factors with 30-day mortality by admission groups according to readmission at day 30

	All admissions			Index admissions only			Day 30 Readmissions only		
	Odds Ratio	95% CI	pvalue	Odds Ratio	95% CI	pvalue	Odds Ratio	95% CI	pvalue
Male	1.30	1.01-1.11	<0.001	1.29	1.15-1.44	<0.001	1.34	1.19-1.51	<0.001
CCI	1.13	1.08-1.18	<0.001	1.13	1.08-1.18	<0.001	1.10	1.05-1.15	<0.001
IMDS	1.01	1.00-1.01	<0.001	1.01	1.00-1.01	<0.001	1.00	0.99-1.00	0.15
Weekend admission	1.06	1.01-1.11	<0.03	1.04	0.98-1.10	0.20	1.10	1.01-1.20	0.04
Age 1 (youngest)	1.11	1.08-1.13	<0.001	1.10	1.08-1.23	<0.001	1.12	1.06-1.18	<0.001
Age 2	0.96	0.93-0.99	0.02	0.97	0.93-1.00	0.06	0.94	0.89-1.00	0.06
Age 3	0.92	0.73-1.17	0.52	0.94	0.73-1.21	0.62	0.96	0.64-1.43	0.83
Age 4 (oldest)	2.57	1.41-4.69	0.002	2.39	1.28-4.47	0.006	2.53	0.90-7.12	0.08
Date 1 (early year)	0.99	0.99-1.00	0.001	0.99	0.99-1.00	<0.001	0.99	0.99-1.00	0.54
Date 2	1.01	1.00-1.02	0.04	1.01	1.00—1.03	0.02	0.99	0.98-1.02	0.94
Date 3	0.97	0.94-1.00	0.06	0.97	0.94-0.99	0.032	0.99	0.95-1.05	0.97
Date 4 (late year)	1.04	1.00-1.07	0.03	1.04	1.00-1.07	0.028	1.02	0.96-1.07	0.50

Note: CCI: Charlson Comorbidity Index; IMDS: Index of Multiple Deprivation Score; Age spline knots: 8, 52, 70, 81 and 91 years; Date spline knots: 16th January, 31st March, 18th June, 17th September and 15th December.
